# Sensory reactivity, empathizing and systemizing in autism spectrum conditions and sensory processing disorder

**DOI:** 10.1016/j.dcn.2017.05.005

**Published:** 2017-05-18

**Authors:** Teresa Tavassoli, Lucy Jane Miller, Sarah A. Schoen, Jennifer Jo Brout, Jillian Sullivan, Simon Baron-Cohen

**Affiliations:** a School of Psychology and Clinical Language Sciences, University of Reading, Reading, RG6 6AL, UK; b Sensory Therapies And Research (STAR) Institute, Greenwood Village, CO, USA; c University of Colorado Denver, Denver, CO, USA; d Rocky Mountain University of Health Professionals, Provo, UT, USA; e International Misophonia Network (IMRN), Greenwich, CT, USA; f Northeastern University, Boston, MA 02115, USA; g Autism Research Centre, Department of Psychiatry, University of Cambridge, Cambridge, CB2 8AH, UK

**Keywords:** ASC, autism spectrum conditions, AQ, autism spectrum quotient, EQ, empathy quotient, TD, typically developing, SPD, sensory processing disorder, SQ, systemizing quotient, Autism spectrum conditions, Sensory processing disorder, Sensory symptoms, Empathy, Systemizing

## Abstract

Although the DSM-5 added sensory symptoms as a criterion for ASC, there is a group of children who display sensory symptoms but do not have ASC; children with sensory processing disorder (SPD). To be able to differentiate these two disorders, our aim was to evaluate whether children with ASC show more sensory symptomatology and/or different cognitive styles in empathy and systemizing compared to children with SPD and typically developing (TD) children. The study included 210 participants: 68 children with ASC, 79 with SPD and 63 TD children. The Sensory Processing Scale Inventory was used to measure sensory symptoms, the Autism Spectrum Quotient (AQ) to measure autistic traits, and the Empathy Quotient (EQ) and Systemizing Quotient (SQ) to measure cognitive styles. Across groups, a greater sensory symptomatology was associated with lower empathy. Further, both the ASC and SPD groups showed more sensory symptoms than TD children. Children with ASC and SPD only differed on sensory under-reactivity. The ASD group did, however, show lower empathy and higher systemizing scores than the SPD group. Together, this suggest that sensory symptoms alone may not be adequate to differentiate children with ASC and SPD but that cognitive style measures could be used for differential diagnosis.

## Highlights

• A diagnostic confusion exists between ASD and SPD, both being associated with atypical sensory reactivity.
• Our aim was to test whether children with ASC and SPD can be differentiated on sensory symptoms and/or cognitive styles in empathy and systemizing.
• Across groups sensory symptoms and empathy showed a negative correlation with each other.
• Both groups, children with ASC and SPD, showed significantly more sensory symptoms than typically developing children.
• The ASC group showed lower empathy and higher systemizing compared to the SPD group; cognitive styles seem useful for differentiating ASC and SPD.

## 1 Background

The ability of the brain to receive, integrate, and respond to an ongoing stream of external sensory information is critical for adaptive responses to the environment. Individuals with autism spectrum conditions (ASC),^1^ however, often report unusual sensory symptoms such as over-reactivity to sound or touch (Chamak et al., 2008; Grandin, 1996, White and White, 1987). Beyond anecdotal reports, questionnaires such as the Sensory Profile have estimated atypical sensory features in over 90% of children and adults with ASC (Baird et al., 2006, Crane et al., 2009; Dunn et al., 2002; Kern et al., 2007, Kientz and Dunn, 1997, Leekam et al., 2007; Tomchek and Dunn, 2007, Watling et al., 2001; Wiggins et al., 2009). A recent observational study also confirmed sensory reactivity symptoms in over 65% of children with ASC (Tavassoli et al., 2016). The growing interest in sensory processing differences in ASC is reflected by the most recent Diagnostic and Statistical Manual (DSM-5) criteria for the condition, which now include over- and under-reactivity to sensory input as well as sensory craving. According to the new DSM-5, hyper-reactivity, over-reactivity here, is defined as an adverse response to sensory stimuli, hypo-reactivity, under-reactivity here, as an indifference to sensory stimuli and sensory craving as an excessive desire for sensory input (A.P.A., 2013).

Atypical sensory symptoms, such as an adverse response to touch, are not unique to ASC. Sensory over- and under-reactivity are reported across many neurodevelopmental conditions including Obsessive-Compulsive and Related Disorder (OCD) (Dar et al., 2012; Levit-Binnun et al., 2013). A growing number of clinicians also have proposed atypical sensory symptoms in children be categorized with the diagnostic term Sensory Processing Disorder, or SPD, with a number of subtypes within the diagnosis (Miller et al., 2009). SPD, originally conceived as sensory integration dysfunction (Ayres, 1969), is reported to affect between 5% (Ahn et al., 2004) and 16% (Ben-Sasson et al., 2009a, Ben-Sasson et al., 2009b) of the general child population. SPD has been acknowledged in some diagnostic classification guides (Classification:0-3R, 2005), but not others (e.g. the DSM-5). We also use the suggested term of Sensory Processing Disorder (SPD) here to refer to children who have sensory processing difficulties.

Diagnostic confusion exists between ASC and SPD due to the lack of research investigating the distinctness of SPD and because many of their defining symptoms overlap. For example, an “apparent lack of interest in… engaging in social interactions” is part of the diagnostic criteria for the under-responsive subtype of regulation disorders of sensory processing in the DC:0-3R, which is very similar to the DSM-5 criteria for ASC which includes “absence of interest in peers”. Only a few studies have directly compared children with ASC and SPD (Schoen et al., 2008). One study used the Sensory Challenge Protocol, in which children are presented with different sensory stimuli while electrodermal activity is measured, and the Sensory Profile, a parent report questionnaire (Dunn, 1999, Schoen et al., 2009): children with ASC showed significantly lower physiological arousal levels than the SPD group and the SPD group showed significantly higher reactivity in response to sensory stimuli than the ASC group. In addition, the Short Sensory Profile revealed group differences, with both children with ASC and SPD showing more sensory symptoms compared to typical developing children. Examining the differences more closely, children in the ASC group showed more taste/smell reactivity and more sensory under-reactivity compared to the SPD group, while sensory craving behaviors were more common in the SPD group compared to the ASC group (Schoen et al., 2009). Brain-imaging studies have also investigated the differences between SPD and typically developing children and children with ASC, finding white matter abnormalities in children with SPD compared to typically developing children (Owen et al., 2013) and differences in white matter tracts between ASC and SPD (Chang et al., 2014). This more recent study further found that both groups showed less connectivity in sensory related tracts but that only the ASC group showed difficulties in socioemotional-related tracts (Chang et al., 2014). Following these few studies, the first aim of this study was to examine the sensory similarities and differences between children with ASC and SPD using the Sensory Processing Scales Inventory (Schoen et al., 2008).

In addition to sensory symptoms, children with ASC display social and communication difficulties alongside unusual repetitive behavior and restricted interests (A.P.A., 1994, A.P.A., 2013). Sensory symptoms are likely associated with core features of ASC and may underlie some of the deficits associated with the condition e.g. repetitive behaviors (Boyd et al., 2010), as well as some of the strengths e.g. attention to detail (Baron-Cohen et al., 2009). The way in which sensory stimuli from the world around us is perceived has an impact on our behavior and cognition and impairments in how sensation is processed and experienced can lead to varied and multiple problems in daily life and mental health (Ben-Sasson et al., 2013, Dar et al., 2012, Liss et al., 2006). Therefore, a second aim of the current study was to investigate whether children with ASC can be differentiated from SPD by their cognitive styles, specifically in terms of empathy and systemizing.

Empathy comprises the drive to identify another person’s emotions and thoughts (the cognitive component), and the appropriate emotional response (the affective component) (Baron-Cohen, 2008, Chakrabarti and Baron-Cohen, 2006). Systemizing is the drive to analyze or construct rule-based systems, whether mechanical, abstract, or any other type (Baron-Cohen, 2008). Studies have shown that individuals with ASC have the tendency to show a greater drive toward systemizing combined with a lower drive toward empathizing (Baron-Cohen, 2008). Clinical observation of children with SPD suggests they have fewer or less severe social and communication impairments than children with ASC but to our knowledge, these cognitive styles have yet to be examined in the SPD population. Since clinical observation of children with SPD suggests they have fewer, less severe social and communication impairments than children with ASC and are not as strongly attracted to lawful domains, we predicted that SPD children would have average empathy and average systemizing profiles. We also predicted that there would be a relationship between these cognitive profile and sensory symptomatology across groups.

In summary, the goals of this study were to determine if children with ASC can be distinguished from children with SPD based on a) sensory reactivity symptoms and b) cognitive styles, specifically empathy and systemizing. Improved sensory and cognitive phenotyping is an essential first step towards reducing diagnostic confusion between ASC and SPD.

## 2 Methods

Data were collected on-line via two websites: www.cambridgepsychology.com for parents of a child with SPD, and www.autismresearchcentre.com for those with a child with ASC. Both portals led to identical versions of the tests. The SPD group were recruited via the Sensory Processing Disorder Foundation (USA) website. Parents could choose a convenient time to complete the on-line tests, and could log out between tests. The study had approval from the Psychology Research Ethics Committee of the University of Cambridge and the Institutional Review Board at Rocky Mountain University of Health Professions.

### 2.1 Participants

The study included 210 participants, of whom 68 had ASC, and 79 had SPD, and 63 were typically developing children (TD) (see Table 1). Parents completed on-line questionnaires and information concerning their child’s diagnosis, sensory symptoms and cognitive styles, specifically empathy and systemizing. In the ASC group parents had to indicate that their child was given a diagnosis of ASC. To screen for autistic traits the Autism Spectrum Quotient-Child (AQ-Child) was used (Auyeung et al., 2008). Criteria for inclusion into the ASC group were an AQ of 26 and above and a diagnosis of ASC in a recognized clinic by a psychiatrist or clinical psychologist using DSM-IV (1994) criteria. The criterion for inclusion in the SPD and TD group were an AQ of 25 or below (i.e. below the risk cut-off) and no previous diagnosis of ASC. Children who had a comorbid diagnosis of SPD and ASD were excluded from the analysis. For the SPD group, parents indicated if their child ever received clinical evaluations suggesting SPD, or Sensory Integration Disorder. Sensory symptoms were assessed using the Sensory Processing Scale Inventory including questions concerning Sensory Over-Reactivity, Sensory Under-Reactivity, and Sensory Craving. Cognitive styles were assessed using the child version of the Empathy Quotient (EQ) and the Systemizing Quotient (SQ).

**Table 1:** Number, sex ratio and age of participants. Mean scores, respective standard deviations (SD) and significance of group differences are also shown. Abbreviations; ASC = Autism Spectrum Conditions, SPD = Sensory Processing Conditions, TD = Typically Developing.

	ASC	SPD	TD
N (m/f)	68 (57/11)	79 (48/31)	63 (34/29)
Age in years (SD, age range)	8.5 (2.4, 5–15)	7.5 (1.9, 5–12)	7.6 (2.4, 4–15)
AQ (SD)	15.9 (6.5)	19.9 (5)	38.44 (5.3)

### 2.2 Measures

#### 2.2.1 Autism spectrum quotient (AQ)

The child version of the AQ (Auyeung et al., 2008) is a short, 50-item questionnaire measuring autistic traits, with 5 subscales (social skills, attention switching, attention to detail, imagination and communication) (Baron-Cohen et al., 2001). A score of 0 was assigned to the responses ‘definitely agree’ and ‘slightly agree’ and a score of 1 for ‘slightly disagree’ and ‘definitely disagree’. Total scores could therefore range from 0 to 50, with higher scores indicating more autistic traits. Results from the AQ have been replicated cross culturally (Hoekstra et al., 2008; Wakabayashi et al., 2004) and across different ages (Auyeung et al., 2008, Wakabayashi et al., 2007, Wheelwright et al., 2010). The AQ also shows good test-retest reliability (r = 0.78) (Baron-Cohen et al., 2001).

#### 2.2.2 The sensory processing scale

The Sensory Processing Scale (SP Scale, now called the Sensory Processing Three Dimensions Scale (SP3D)) (Miller and Schoen, 2012; Schoen et al., 2008) has two parts: an inventory report-measure, completed by parents, caregivers or self, and a performance measure or assessment, administered by an examiner. Only the inventory was administered in this study, specifically the subscales regarding Sensory Under-Reactivity (SUR; e.g., *Typically my child does not notice strong odors;* 30 items), Sensory Over-Reactivity (SOR; e.g., *These smells bother my child, e.g. soap;* 76 items), and Sensory Craving (SC; e.g., *My child has a constant desire for swinging;* 37 items). The SP Scale reflects sensory reactivity including over-reactivity, under-reactivity and sensory craving across all sensory domains (tactile, visual, olfactory, auditory, vestibular, proprioception and gustatory). Previous research on the Sensory Over-Reactivity (SOR) subscale showed high internal consistency reliability within each domain (Cronbach’s a = 0.65–.88; (Schoen et al., 2008)). In addition, the SOR inventory has strong discriminant validity, distinguishing between individuals with and without SOR within each domain (*p <* 0.05–.001) and strong concurrent validity with the sensory sensitivity and sensory avoiding dimensions of the Sensory Profile (*r* = 0.47, *p <* 0.01) (Schoen et al., 2016, Schoen et al., 2008). Cronbach’s alpha levels ranged from 0.69 to 1.00 and intraclass correlation coefficients ranged from 0.82 to 1.00 (Lane et al., 2010). All have been shown to differentiate between individuals with and without sensory problems. Each item is scored as a ‘1’ if the parent ticks yes on the item. The number of questions on each Inventory varies by subscale: SOR = 76 items, SUR = 30 items, SC = 37 items. Total scores are then computed for each subtype, with higher scores reflect a greater number of atypical sensory symptomatology.

#### 2.2.3 Empathy quotient (EQ) and systemizing quotient (SQ)

The child version of the EQ and SQ were used (Auyeung et al., 2009). The 27 EQ items measure how easily the child can pick up on other people's feelings and how strongly they are affected by other people's feelings (e.g. “*My child likes to look after other people.”* “*My child is often rude or impolite without realising it”)*. The 28 SQ items assess the child’s interest in systems (e.g. *My child is interested in understanding the workings of machines (e.g. cameras, traffic lights, the TV, etc.*”). Together these are assessed on a single 55-item questionnaire, the child EQ-SQ. The parent is asked to indicate how strongly they agree with each statement as a description of their child. Response options are the following: ‘definitely agree’, ‘slightly agree’, ‘slightly disagree’, or ‘definitely disagree’. Both agree responses are scored as 0, and both disagree responses are a 1, with some items reverse-scored and the items summed by subscale. Higher scores indicate a greater empathizing or systemizing drive. The test-retest reliability of this scale is high (ICC = 0.86) (Auyeung et al., 2009).

## 3 Results

The statistical software package SPSS 20 was used to analyze the data. To correct for multiple comparisons, Bonferroni corrections were used. There was no significant difference in age between groups (p > 0.05). The ASD group had significantly higher scores on the AQ compared to the SPD and TD group (p = 0.0001). The SPD group had a significantly lower AQ score compared to the ASD group (p = 0.0001) and a significantly higher AQ score than the TD group (p = 0.0001).

### 3.1 Sensory symptoms

**Fig. 1:**
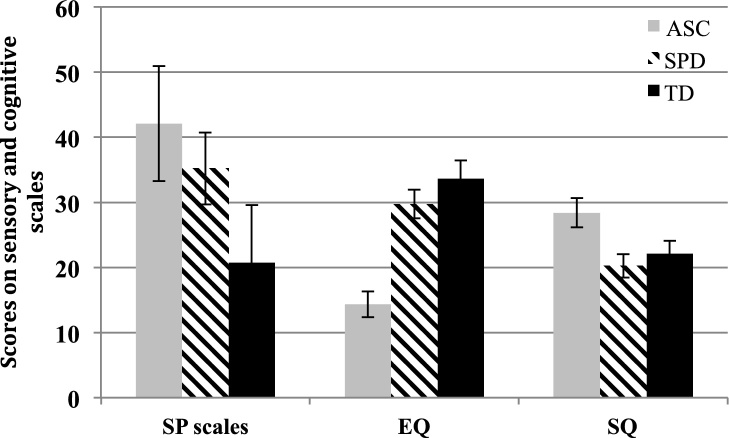
Sensory and cognitive symptoms in children with ASC, SPD and TD. The bars represent combined sensory processing scale (SP scale) scores, Empathy (EQ) and Systemizing Quotient (SQ) scores for children with ASC (Autism Spectrum Conditions), children with Sensory Processing Disorder (SPD), and typical developing children (TD). Error bars represent 95% confidence intervals for the mean. On the SP Scale, high indicates greater impairment. On the EQ high indicates more empathy, and on the SQ a high score indicates greater systemizing. All groups differed on sensory symptoms and empathy. Children with ASC showed highest sensory symptoms, lowest empathy and highest systemizing scores compared to children with SPD and TD children. Children with SPD and TD did not differ in regards to systemizing.

To analyze sensory symptoms, a MANOVA was performed with group as fixed factor and all sensory subscales (Sensory Under-Reactivity, Sensory Over-Reactivity and Sensory Craving) as dependent variables. Using Pillai’s trace, there was a significant effect of group on the amount of atypical sensory behaviors (F (3,190) = 9.0, *p <* 0.0001) (see Table 2). Post hoc pairwise comparisons were next conducted to explore group-level differences. For the SUR subscale, the ASD group scored higher than the SPD group (p = 0.02), who in turn scored higher than the TD group (p = 0.01). On the SOR subscales, the ASC and SPD groups did not significantly differ from one another (p = 0.19), but both scored significantly higher than the TD group (p < 0.01). Both children with ASC and SPD also showed higher scores on Sensation Craving compared to TD children (*p *< 0.01), but did not differ from each other (*p* = 0.99) (Fig. 1).

**Table 2:** Sensory Processing (Sensory Over-Reactivity/SOR, Sensory Under-Reactivity/SUR and Sensory Craving), Empathy Quotient (EQ) and Systemizing Quotient (SQ) scores in children with ASC, SPD and typical developing children (TD). Mean scores, respective standard deviations and significance of group differences are also shown.

	SOR	SUR	Craving	EQ	SQ
***ASC***	***22.9*** ***(12.6)***	***8.2*** ***(5.0)***	***10.9*** ***(7.0)***	***14.3*** ***(8.3)***	***28.4*** ***(9.4)***
boys	24.9 (12.4)	8.6 (5.1)	11.5 (7.2)	13.4 (7.0)	28.3 (9.1)
girls	13.9 (9.0)	6.7 (3.9)	8.0 (5.8)	20.5 (12.5)	28.3 (7.6)
***SPD***	***19.2*** ***(9.4)***	***5.5*** ***(4.3)***	***10.4*** ***(6.7)***	***29.7*** ***(9.9)***	***20.2*** ***(8.1)***
boys	20.6 (8.6)	5.7 (4.1)	11.5 (6.0)	28.0 (9.3)	20.6 (8.6)
girls	17.2 (10.3)	5.2 (3.9)	8.7 (7.4)	32.0 (10.6)	17.2 (10.3)
***TD***	***11. 8*** ***(12.7)***	***3.3*** ***(4.3)***	***5.4*** ***(6.5)***	***33.6*** ***(4.2)***	***22.11*** ***(8.1)***
boys	10.5 (10.9)	3.9 (4.7)	5.1 (6.9)	31.9 (11.3)	22.1 (7.3)
girls	13.3 (14.5)	2.7 (3.7)	5.8 (6.3)	34.7 (12.1)	21.2 (7.3)
Group Difference F score (p)	14.1 (0.0001)	17.0 (0.0001)	11.5 (0001)	60.1 (0.0001)	14.8 (0.0001)

### 3.2 Empathy and systemizing

Regarding cognitive profiles, the EQ and SQ scores for typical developing children were in the average range as reported by Auyeung et al. (2009). An MANCOVA was conducted with group as fixed factor and EQ and SQ as the dependent variables. Sex was entered as a covariate, since there is a reported sex difference in EQ scores for typical developing children (Auyeung et al., 2009). Using Pillai’s trace, there was a significant effect of group (F (2, 205) = 31.3, *p *< 0.0001) and sex (F = 5.4, *p* = 0.005) on EQ and SQ scores. Tests of between-subject effects showed that groups differed on the EQ and SQ scores (see Table 2 and Fig 1). Sex had an effect on EQ scores (F = 7.9, *p* = 0.005), girls scoring higher than boys, but not on SQ scores (F = 1.6, *p* = 0.20). Children with ASC showed lower EQ scores compared to children with SPD (*p <* 0.001) as well as TD children (p < 0.001). Children with SPD scored marginally lower than TD children on the EQ (*p* = 0.06). Children with ASC scored higher than both other groups on the SQ (*p* = 0.001), children with SPD and typical developing children showing similar mean scores (*p* = 0.60).

### 3.3 Correlations

**Fig. 2:**
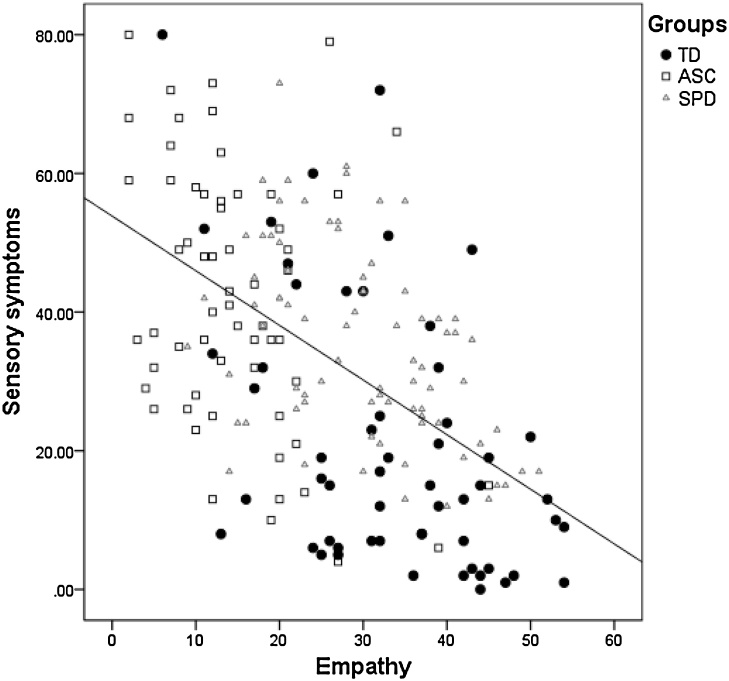
Correlation between sensory and empathy in typically developing children (TD) and children with Autism Spectrum Disorder (ASC) and Sensory Processing Disorder (SPD). Higher empathy scores were correlated with fewer sensory symptoms.

Correlations were calculated between EQ, SQ and all sensory scales combined (Sensory Total, maximum score of 143). Across groups, the EQ score was negatively correlated with the Sensory Total score (r = −0.52, p = 0.01), as well as within each group independently (ASC: r = −0.33, *p* = 0.001; SPD: r = −0.46, *p*=0.001; TD: r = −0.48, *p* = 0.001). In other words, individuals with higher empathy scores had fewer sensory symptoms (see Fig. 2). The SQ was not correlated with total sensory symptoms in any group nor across the groups.

The AQ was correlated with total sensory symptoms in the SPD (r = 0.52, *p* = 0.001), and TD (r = 0.57, *p* = 0.001) groups, suggesting that greater autistic symptomatology is associated with more atypical sensory symptoms, but this did not hold in the ASC group (r = 0.20, *p* = 0.12).

## 4 Discussion

Sensory reactivity is a new DSM-5 criterion for Autism Spectrum Conditions (ASC). However, children who do not have ASC can also suffer from sensory reactivity symptoms as well—children with the suggested diagnostic term of Sensory Processing Disorder (SPD). The current study tested whether there are sensory and/or cognitive features that distinguish ASC from SPD. Children with ASC *or* SPD showed more sensory symptoms than typical developing children, as predicted. The ASC group was the most affected group overall, showing significantly greater symptoms of sensory under-reactivity than both the TD and SPD groups, although they did not differ from the SPD group on sensation craving or sensory over-reactivity symptomatology. Thus, given the overlap in sensory symptoms in ASC and SPD, sensory symptoms alone are not adequate to differentiate these two groups.

In terms of cognitive style, children with ASC had difficulty in empathy alongside good systemizing skills, versus children with SPD, who had lower systemizing skills but greater empathy compared to children with ASC. Typical developing children had no heightened sensory symptomatology and average levels of parent-reported empathy and systemizing. Children with SPD also had average levels of empathy and systemizing. This suggests that empathy and systemizing are useful cognitive dimensions for differentiating ASC from SPD and has implications for improving diagnostic accuracy, especially for the new DSM-5.

Taken together children with ASC showed the greatest sensory symptomatology and lowest empathy. Children with ASC showed lower parent-reported empathy compared to children with SPD. In children with ASC the underlying disability to empathize may explain the social and communication difficulties (Baron-Cohen, 2008, Baron-Cohen et al., 2002). Given that in our current study individuals with higher empathy scores had fewer sensory symptoms, difficulties of understanding others might also impact the amount of sensory symptoms in children with ASC or vice versa. Children with SPD, who have not been characterized on empathy beforehand, had slightly lower empathy scores than typically developing children. In corroboration, while children with SPD in the current study scored below the cut-off on the AQ, they also had significantly higher scores compared to TD children. Indeed, therapists and parents have reported that children with SPD often have difficulty in the behavioral and emotional domains, particularly with regard to emotion regulation (Miller, 2006). When barraged by sensations that others would not notice such as a loud shopping mall, a child who is over-reactive to sensory stimuli might for example feel overloaded and exhibit dysregulated behavior. By the time a child with SPD enters school, relationships may be compromised and they may present with emotional and behavioral problems. Consequently, empathy may be impaired in SPD because these challenges make it difficult to respond appropriately to another person’s emotions. Future studies are needed to test if and how sensory reactivity problems affect social cognition and behavior or might represent a risk factor regarding establishing healthy foundation for emotional development, early relationships, and emotional maturity.

Furthermore, the total numbers of sensory symptoms and social features were associated with one another across groups, specifically with greater sensory symptoms predicting lower parent-reported empathy. Even though children with SPD had augmented empathy scores compared to children with ASC, their scores were lower than the TD group. The association between sensory perception and social cognition is long known. In an early stage of development, infants seek physical contact and learn via their senses to form an attachment to their caregiver. Bowlby, 1958, Bowlby, 1969, Bowlby, 1988, Bowlby, 1989 argued that through attachment, the infant develops mental representations that become templates for future relationships. However, attachment models do not take into consideration the dysregulating effect of atypical sensory reactivity. An effective and appropriate reaction to sensory stimulation, such as speech sounds, visual facial cues, and social touch, is especially important in order to attend to and decipher social cues and respond flexibly (Ben-Sasson et al., 2009a, Ben-Sasson et al., 2009b). Future studies should investigate what effect sensory reactivity issues have on social skills, attachment and later development.

Limitations of this study include that it was a self-selected sample and data was collected online. Using an online survey allowed us to collect data from a larger group of participants, but lacks some control over variables and a laboratory study including an IQ measure is needed to test if the current findings can be duplicated. However, online data collection does confer the advantage of increasing diversity and minimizing experimental bias, and numerous studies have shown that online survey methodology and data are at least equivocal or even better in quality than performing the study in a traditional laboratory setting (e.g., (Buchanan and Smith, 1999, Riva et al., 2003)). In addition, it would be important to test if children with SPD can be differentiated from children with other conditions such as Attention Deficit Hyperactivity Disorder (ADHD), OCD, or anxiety. Here, children with additional conditions were excluded from this study. Future research is needed to distinguish sensory symptoms in children with SPD from other childhood disorders such as ADHD. Recent work suggests that sensory symptoms differ in children with SPD and ADHD (Yochman et al., 2013).

The current findings are also worth further exploration using behavioral and performance-based tasks, which measure sensory reactivity and empathy. It would also be interesting to compare children with ASC, SPD and TD children on sensory and social tasks using neuroimaging. A recent DTI brain imaging study showed that both children with ASC and SPD had decreased connectivity relative to TD children in white matter tracts involved in sensory perception (Chang et al., 2014). However, only the ASD group showed decreased connectivity compared to TD children in tracts related to social processing. This suggest that even though sensory reactivity is affected in both groups on a behavioral and biological basis, social processing likely seems to be intact at least on a biological basis for children with SPD. This has direct implications for different treatment recommendations for children with ASC and SPD.

## 5 Conclusions

This study sheds light on the similarities and differences between children with ASC and SPD, which could be helpful for distinguishing these two conditions. Taken together, our findings show that children with ASC are most affected by sensory symptoms, and show lowest empathy and highest systemizing scores. Scores for children with SPD fall in between those for children with ASC and typical developing children on these measures. Future longitudinal studies are needed to explore if children with ASC and SPD both start with the same amount or type of sensory symptoms in early childhood and whether there is a difference in the type of sensory symptoms they display. Children with ASC also have the greatest difficulties in empathy, which could lead to more severe overall symptoms. Children with SPD on the other hand might have an intact drive to empathize, but sensory issues might stop them from using these skills as much as typical developing children. Gathering as much information as possible by measuring cognitive profiles as well as sensory symptoms allows a broader characterization of each child. Identifying greatest areas of challenges, being low empathy or heightened sensory reactivity, can guide treatment. Future work is needed to validate these results using performance tests and to understand the neural basis of the similarities and differences between these two related conditions.

## Conflict of Interest

None.

## Footnotes

1 DSM-5 uses the term ‘Autism Spectrum Disorders’ (ASD) but we opt to use the less stigmatizing term ‘Autism Spectrum Conditions (ASC)’ which is now increasingly used in Europe. We have not amended the term ‘disorder’ in relation to SPD because the recognition of SPD is still too new and this still requires the relevant discussions to be had within that community.
